# Infralimbic GABAergic May be the Target of Asiaticoside on Alleviating Bone Cancer Pain

**DOI:** 10.1002/brb3.70555

**Published:** 2025-05-19

**Authors:** Xin Li, Jiayu Tong, Xinru Yuan, Xiaoxuan Zhang, Haonan Yu, Chunlei Xing, Jingxiang Wu, Yuwei Qiu, Xingji You

**Affiliations:** ^1^ School of Medicine Shanghai University Shanghai China; ^2^ Department of Anesthesiology, Shanghai Chest Hospital Shanghai Jiao Tong University Shanghai China

**Keywords:** asiaticoside, bone cancer pain, GABAergic, infralimbic cortex

## Abstract

**Purpose:**

Bone cancer pain (BCP), characterized by neuropathic and inflammatory components due to bone metastasis and immune responses, remains a significant clinical challenge. Asiaticoside (AS), an active compound derived from *Centella asiatica*, exhibits diverse pharmacological properties and holds promise for BCP treatment. In this study, we investigated the mechanisms underlying AS‐mediated pain relief.

**Methods:**

By analyzing the expression of the activity‐induced immediate‐early gene c‐Fos and performing whole‐brain mapping to identify activated regions after the treatment of AS. Chemogenetic approaches were employed to selectively activate or inhibit glutamatergic and GABAergic neurons in the IL region.

**Results:**

Our findings revealed that the infralimbic cortex (IL) plays a critical role in AS‐induced analgesia. Further analysis demonstrated that GABAergic neurons, rather than glutamatergic neurons, were predominantly activated in the IL region, suggesting their involvement in pain alleviation. Moreover, AS significantly alleviated BCP by activating GABAergic neurons, while their inhibition attenuated the analgesic effect. In contrast, modulation of glutamatergic neurons had no significant impact on pain relief. These findings indicate that GABAergic neurons in the IL are essential for the antinociceptive effects of AS.

**Conclusions:**

In conclusion, our study demonstrates that AS alleviates BCP by selectively targeting GABAergic neurons in the infralimbic cortex, providing a potential therapeutic strategy for managing BCP. This work highlights the importance of GABAergic signaling in pain modulation and offers new insights into the development of targeted therapies for cancer‐induced pain.

## Graphical abstract

Our study demonstrates that AS alleviates BCP by selectively targeting GABAergic neurons in the infralimbic cortex (IL). These findings highlight the pivotal role of IL GABAergic signaling in pain modulation. Furthermore, this study provides preclinical evidence supporting AS to be a promising pharmacological agent with a targeted delivery strategy for clinical pain intervention. Further research is needed to elucidate the downstream neural circuits involved in AS‐mediated analgesia.

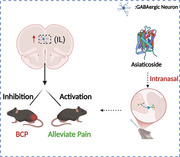

## 1 Introduction

The incidence of cancer has been increasing in the past 10 years (Miller et al. 2020), and in the late stage of a variety of cancers, bone metastasis‐induced cancer pain (BCP) occurs accompanied by cognitive impairment and negative emotions which severely impacts the patient's quality of life (Frost et al. 2016). While the typical treatment measures used to reduce, alleviate, or manage pain sensations through the consumption of over‐the‐counter NSAIDs and prescription opioids may accompany low long‐term benefits and certain risks, new drugs with low side effects are urgent to be found.

Asiaticoside is a triterpene found in Centella, most recently, it has been reported that exhibits neuroprotective, cognition‐enhancing anti‐cancer chemotherapeutic and chemo‐preventive activities via multi‐targets and multi‐pathways (Zhang et al. 2020; Kwon et al. 2014; Chen et al. 2014; Al‐Saeedi 2014). For instance, in cerebral ischemia‐reperfusion injury, asiaticoside provides a protective effect via the NOD2/MAPK/NF‐κB signaling pathway (Zhang et al. 2020). In addition, asiaticoside regulates the PI3K‐Akt‐mediated inflammatory pathway such as attenuating diabetes‐induced cognition deficits in the hippocampus (Yin et al. 2015), and alleviating cardiomyocyte apoptosis (Zeng et al. 2022). Importantly, asiaticoside also has been evidenced to exert beneficial effects on attenuating pain symptoms after spinal cord injury (Luo et al. 2015; Ayumi et al. 2020). Moreover, a previous study showed that asiaticoside has preventive effects in experimental migraine (Bobade et al. 2015), which indicated that asiaticoside may exhibit an effective analgesic response. However, the exact pharmacological effect of asiaticoside on bone cancer pain (BCP) has not been clarified.

The emotional and cognitive components of pain are processed by the medial prefrontal cortex, which comprises the anterior cingulate cortex (ACC), prelimbic cortex (PrL), and infralimbic cortex (IL) (Kummer et al. 2020; Kiritoshi et al. 2016). In chronic pain states, the IL region gradually diminishes (Ma et al. 2024b; Cui et al. 2024;Ma et al. 2024a; Leite Ferreira et al. 2022), which suggests a strong connection between the IL region and chronic pain. Glutamatergic outputs and GABAergic signaling are particularly emerging as relevant components of affective pain processing within the IL (Cui et al. 2024). Activation of pyramidal neurons in the IL alleviates LPS‐induced depressive‐like behavior in mice (Zhang et al. 2024). However, the activity of IL neurons and their role in BCP remains elusive.

In this study, asiaticoside was used to treat cancer‐induced bone pain (CIBP) in a C57BL/6 mice model, and we found that the pain was relieved after the treatment. In addition, to pinpoint the specific cell populations responsible for the alleviated cancer pain by asiaticoside, we use immunohistochemistry to assess the expression of the activity‐induced immediate‐early gene, c‐Fos, 120 min after asiaticoside injection of cancer pain established D14. With an emphasis on the IL, the present study revealed that asiaticoside is a positive modulator of GABAergic neurons in IL to alleviate cancer pain. In summary, the current study analyzed the pharmacological mechanisms of BCP in asiaticoside treatment.

## 2 Methods

### 2.1 Animals and Ethics Statement

In the present study, C57BL/6J, VGluT2‐Cre, and VGAT‐cre mouse lines at the age of 8–12 weeks old were used for experiments and housed 5/cage under 12‐hour light‐dark cycle at the temperature of 24 ± 1°C, with food and water ad libitum access. All experimental procedures complied with the International Association for the Study of Pain guidelines and were approved by the Animal Care and Use Committee of Shanghai Chest Hospital, Shanghai Jiao Tong University.

### 2.2 BCP Mice Model

Murine lung carcinoma cell line LLC was digested with 0.05% trypsin and made into a suspension of 2 × 10^7^/mL cells in PBS. The inoculation was performed as previously described (Zhang et al. 2025). The animals were anesthetized with Tribromoethanol, and a 0.5–1 cm superficial incision was made near the knee joint to expose the patellar ligament. Then a 25‐gauge needle was inserted at the site of the intercondylar notch of the right femur into the femoral cavity, and the needle was then replaced with a 10 µL microinjection syringe containing a 10 µL suspension of tumor cells (2 × 10^5^). The contents of the syringe were slowly injected into the femoral cavity. To prevent leakage of tumor cells outside the bone cavity, the outside injection site was sealed with fibrin glue or movement dysfunction after surgery was excluded.

### 2.3 Administration Method

Asiaticoside (HY‐N0439, MCE) was administered at doses of 60 or 120 mg/kg, dissolved in PF‐127 (P2443, Merck). A volume of 20 µL per nostril was typically used, ensuring that the volume was not excessive to prevent aspiration into the lungs. Using a micropipette (such as a 20 µL pipette) or a fine dropper, the liquid is slowly instilled into one or both nostrils. After administration, the animal was allowed to inhale spontaneously, and its head was maintained in a tilted position for 30 s–1 min to prevent the liquid from flowing out or entering the digestive tract.

Tamoxifen (HY‐13757A, MCE) was injected intraperitoneally at a dose of 150 mg/kg.

### 2.4 Pain Behavior Test

As previously reported (Lu et al. 2019), the von Frey filaments were used to determine the paw withdrawal threshold of responding to mechanical stimuli. Briefly, mice were individually placed in a separate compartment with a metal mesh floor. After the mouse adapted for about 30 min, von Frey filaments were used vertically to stimulate the plantar surface of the right hind paw for 3∼5 s. Ascending order of force is used throughout the process (0.008, 0.02, 0.04, 0.07, 0.16, 0.4, 0.6, 1.0 g), starting at 0.008 g and ending at 1 g. Paw withdrawal, shaking, and licking were considered to be positive responses. PWT was defined as the minimum force (in grams) required to elicit positive responses. The whole experiment was conducted by researchers who were blind to the group settings.

Under stable room conditions of temperature and humidity, all behavioral tests took place in individual plexiglass chambers on metal mesh floors. The mechanical paw withdrawal frequency (PWF) of each mouse group was measured. Before testing the pain behavior of mice, they need to acclimate in the behavioral room for 60 min. Von Frey filaments of 0.16 g were applied to the plantar surface of the hind paw at 10‐s intervals. The filament was considered to have bent into an S shape for a duration of ≤ 6 s, with 10 repetitions. A sudden retraction or paw filling was indicative of a positive reaction. Frequency is based on the percent of paw withdrawal following each filament stimulus.

### 2.5 AAV Injection

AAV serotype (AAV2/9) was diluted to working concentration using sterile PBS, aliquoted, and stored at −80°C in light‐proof conditions. AAV2/9‐hSyn‐CRE‐mCherry, AAV2/9‐hSyn‐DIO‐hM4Di‐mCherry, AAV2/9‐hSyn‐DIO‐hM3Dq‐EGFP, AAV2/1‐Vgat‐cre‐EGFP were purchased from BrainVTA, AAV2/9‐ESARE‐ERT2‐Cre were purchased from shanghai Taitool Bioscience. The virus was thawed on ice prior to injection. Mice were anesthetized with tribromoethanol and secured in a stereotaxic apparatus (RWD, China). The surgical area was disinfected with iodophor followed by 75% ethanol. A burr hole was drilled in the skull and IL: (AP: −2.5 mm, ML: ±0.5 mm, DV: −3.0 mm). A microinjection pump (Hamilton syringe, 33G needle) was used to deliver the viral suspension at a constant flow rate (50 nL/min). The total injection volume per unilateral/bilateral site was 150 nL. The needle remained in place for 5 min post‐injection to prevent backflow, then was slowly withdrawn. The skin was sutured afterward. Animals were placed on a heating pad for recovery and monitored for at least 24 h. They were provided access to food/water and analgesic support.

### 2.6 Immunofluorescence Staining

Brain slices were fixated in 4% paraformaldehyde (PFA), permeabilized, and blocked using PBS containing 5% BSA and 0.5% TritonX‐100. Primary antibody anti‐c‐Fos (1:1000, oasis, China), Anti‐VGLUT2(1:500, Abcam, USA), Anti‐GAD65(1:500, abconal, China) was incubated overnight at 4°C with brain slices. The slices were then incubated with Alexa Fluor 594‐conjugated Goat Anti‐Guinea Pig IgG (H+L) Cross‐Adsorbed Secondary Antibody (1:2000; Invitrogen; United States) and Alexa Fluor 594 conjugated anti‐Rabbit IgG secondary antibody (1:2000, Invitrogen, USA) for 2 h, followed by staining with DAPI (Beyotime, China). The images were visualized by confocal laser microscopy (Olympus, VS200, Japan).

### 2.7 H&E Staining

Tibial bone tissues from the tumor‐inoculated side of BCP model mice and age‐matched sham‐operated controls were harvested at the experimental endpoint (e.g., 14 days post‐inoculation). Tissues were fixed in 4% PFA for 48 h, followed by decalcification in 10% EDTA (pH 7.4) for 14 days at 4°C. Decalcified bones were dehydrated through a graded ethanol series, embedded in paraffin, and sectioned into 5‐µm‐thick slices using a microtome. Sections were deparaffinized in xylene, rehydrated in a descending ethanol series, and stained with hematoxylin (Sigma‐Aldrich, H9627) for 5 min. After rinsing in tap water, sections were differentiated in 1% acid alcohol and blued in 0.2% ammonia water. Counterstaining was performed with eosin (Sigma–Aldrich, E4382) for 2 min, followed by dehydration and mounting with neutral balsam. Histopathological changes (e.g., bone structure destruction, tumor cell infiltration) were visualized under a light microscope (Nikon Eclipse E100) and quantified using ImageJ software (NIH) by measuring trabecular bone area or osteolytic lesion size.

### 2.8 TRAP Staining for Osteoclast Detection

Tibial bone tissues from CIBP model mice and sham‐operated controls were collected at day 14 post‐tumor inoculation. Tissues were fixed in 4% PFA for 24 h, decalcified in 10% EDTA (pH 7.4) for 7 days at 4°C, and embedded in paraffin. Serial 5‐µm‐thick sections were deparaffinized, rehydrated, and incubated with TRAP staining solution (0.1 M sodium acetate buffer, pH 5.0, containing 0.01% naphthol AS‐MX phosphate, 0.06% Fast Red TR salt, 50 mM sodium tartrate, and 0.1% Triton X‐100) for 1 h at 37°C in the dark. Sections were counterstained with hematoxylin for 30 s, rinsed, and mounted with an aqueous medium. TRAP‐positive multinucleated osteoclasts (≥3 nuclei, bright red cytoplasmic staining) adjacent to bone surfaces were quantified as osteoclast number per bone perimeter (Oc.N/B.Pm) using ImageJ software (NIH) under a light microscope (Nikon Eclipse E100).

### 2.9 Data Analysis and Statistics

All data were shown as the standard error of the mean (Mean+SEM). For the data meeting, normal distribution and homogeneity of variance, differences among the groups were analyzed using one‐way analysis of variance (ANOVA) followed by Tukey's multiple comparison test. Two‐way ANOVA followed by Tukey's multiple comparisons test with repeated measurements was used to analyze the differences in latency among groups. The behavior test was repeated 3 times, and *p* < 0.05 was considered statistically significant. All statistical analyses were performed by GraphPad Prism 8.0 for Windows (San Diego, CA, USA), and statistical significance was determined as *p* < 0.05.

## 3 Results

### 3.1 The Mouse Model of BCP Established

**FIGURE 1:**
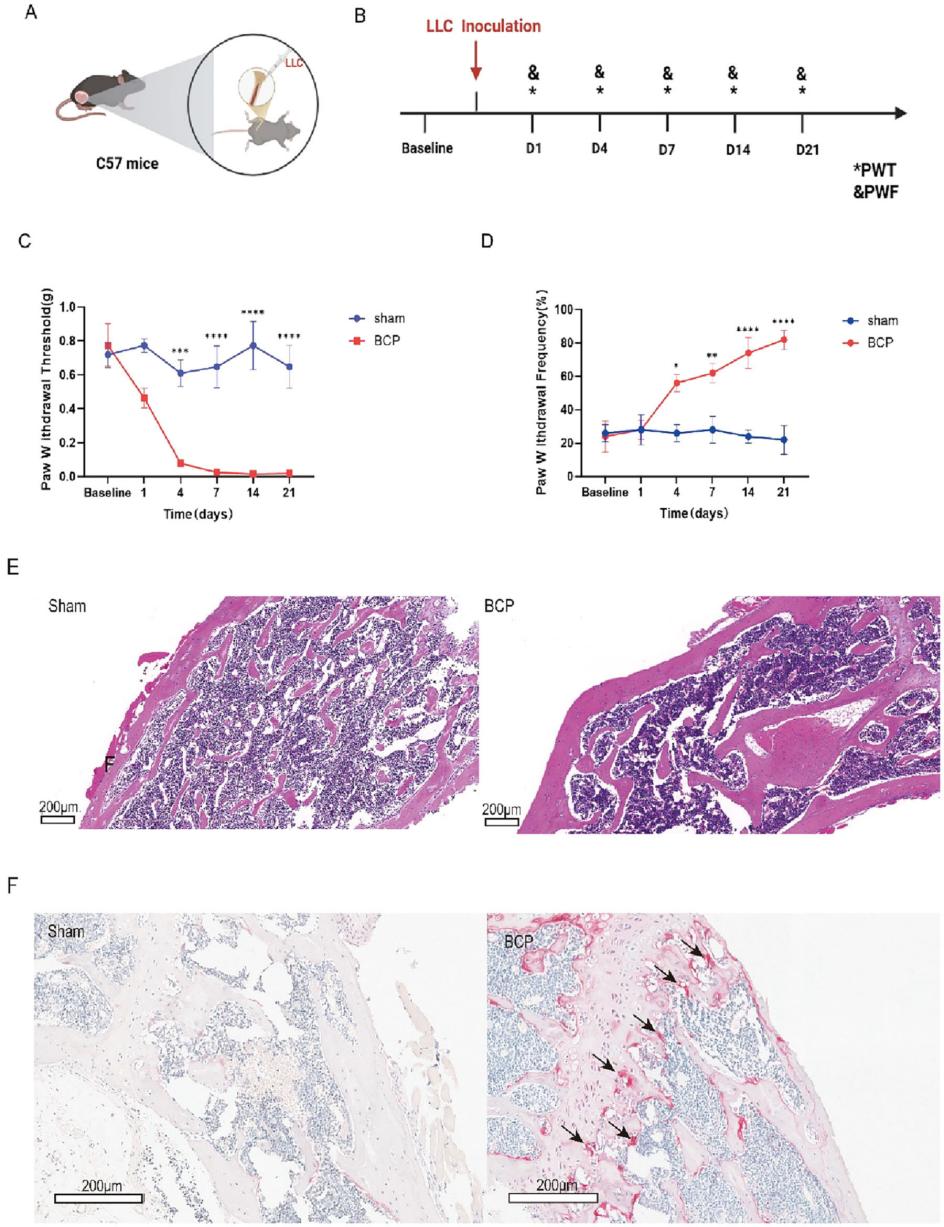
**LLC inoculation can induce pain in a mouse model**. (A) Experimental diagram showing LLC inoculation (B) behavior test and tumor injection administration time. (C) The mice paw withdrawal thresholds (PWT) (D) The mice paw withdrawal frequency (PWF), (E) HE staining of tibial on tumor inoculated side are used to mark the areas of bone destruction at day 21 in the BCP rats. (F) TRAP staining of osteoclasts in bone tissue sections. Representative images of TRAP‐positive osteoclasts (red/purple staining, black arrows) in the tibial bone marrow cavity of Sham group and BCP group. Scale bar: 200 µm. *p < 0.05, **p < 0.01 BCP versus sham group. Error bars are represented as mean ± SEM. *n* = 6 per group.

First, we established a model of CIBP by injecting LLC tumor cells into the femur of the mouse. The behavioral indicators, including PWT and PWF, were tested on Days 0 (baseline), 1, 4, 7, 14, and 21. Figure 1A,B illustrates the experimental procedure for animal testing and the measurement method of PWT and PWF. The results showed that the BCP group had a significant reduction in paw withdrawal threshold (Figure 1C) and PWF (Figure 1D). Compared to the Sham group, which exhibited intact trabecular bone architecture and homogeneous medullary cavities, the BCP group demonstrated marked osteolytic destruction characterized by trabecular fragmentation and neoplastic cell infiltration (Figure 1E). Furthermore, TRAP staining revealed a substantial accumulation of TRAP‐positive multinucleated osteoclasts (black arrows) along the bone surface in the BCP cohort, with significantly elevated activity. These hyperactivated osteoclasts were spatially correlated with bone resorption pits (Howship's lacunae, dashed lines), suggesting that aberrant osteoclast activation underlies pathological bone degradation and nociceptive sensitization in the BCP model (Figure 1F).

### 3.2 Asiaticoside Alleviates BCP

**FIGURE 2:**
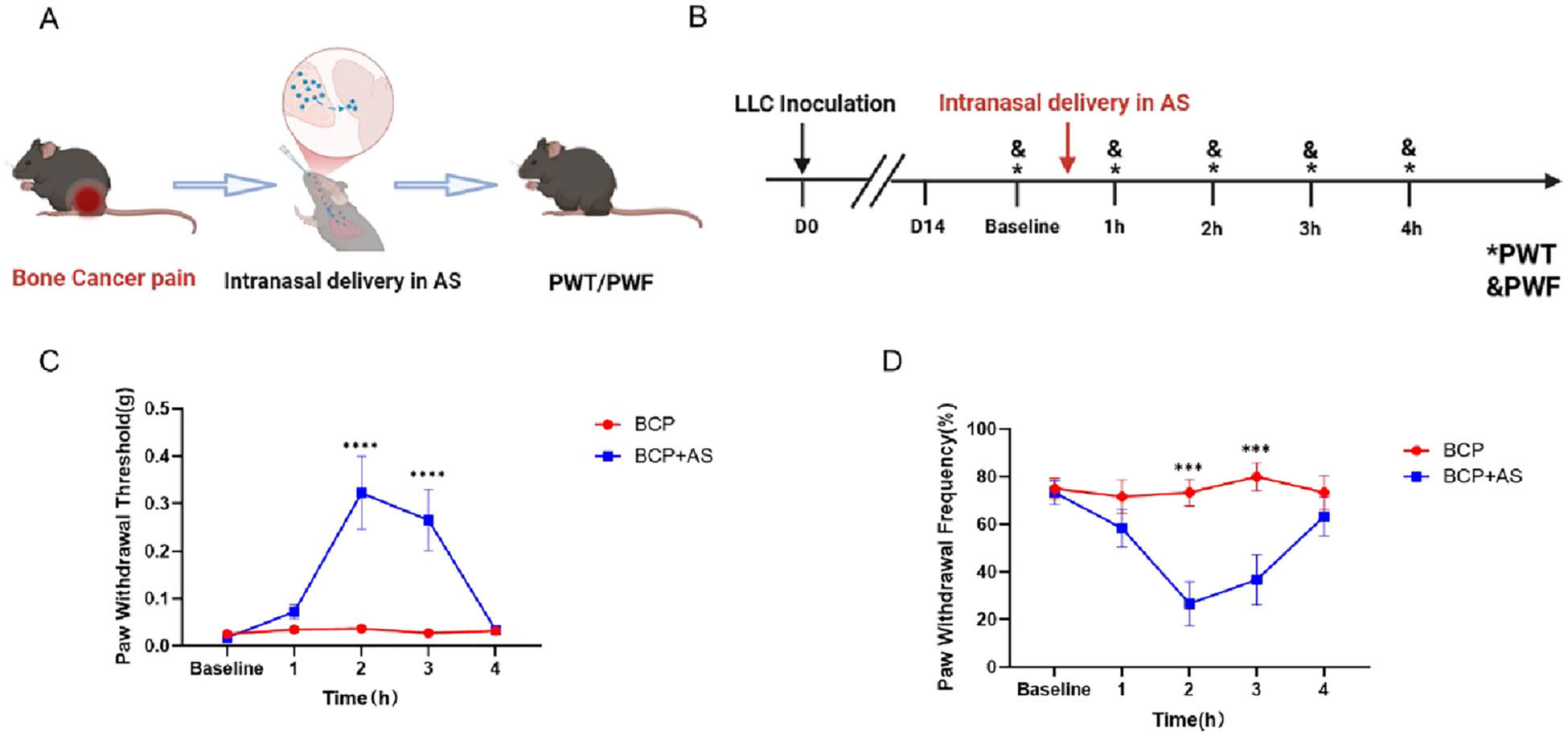
**Asiaticoside alleviates bone cancer pain**. (A) Experimental diagram showing the administration of asiaticoside can alleviate the cancer induced bone pain. (B) Behavior test and intranasal administration time. (C) The mice paw withdrawal thresholds (PWT) under the asiaticoside administration at the D14 after the tumor injection. (D) Paw withdrawal frequency (PWF) under the asiaticoside administration at the D14 after the tumor injection. ***p < 0.001, ****p < 0.0001 BCP versus BCP+AS group, Error bars are represented as mean ± SEM. *n* = 6 per group.

Evidently, asiaticoside has been evidenced to exert beneficial effects on attenuating pain symptoms (Luo et al. 2015; Ayumi et al. 2020; Bobade et al. 2015), To figure out whether asiaticoside can alleviate CIBP, we checked paw withdrawal thresholds after intranasal administration of asiaticoside (Figure 2A,B). Worthwhile, AS administration at 60 and 120 mg/kg significantly elevated mechanical pain thresholds and 120 mg/kg demonstrated enhanced analgesic potency (Figure S1). Then, a dose of 120 mg/kg was selected for subsequent experiments. Notably, intranasal AS delivery demonstrated rapid pain relief in BCP models, achieving significant mechanical threshold elevation within 2 hours post‐administration (Figures 2C‐2D).

### 3.3 An Asiaticoside‐Responsive Antinociceptive Ensemble in the BCP

**FIGURE 3:**
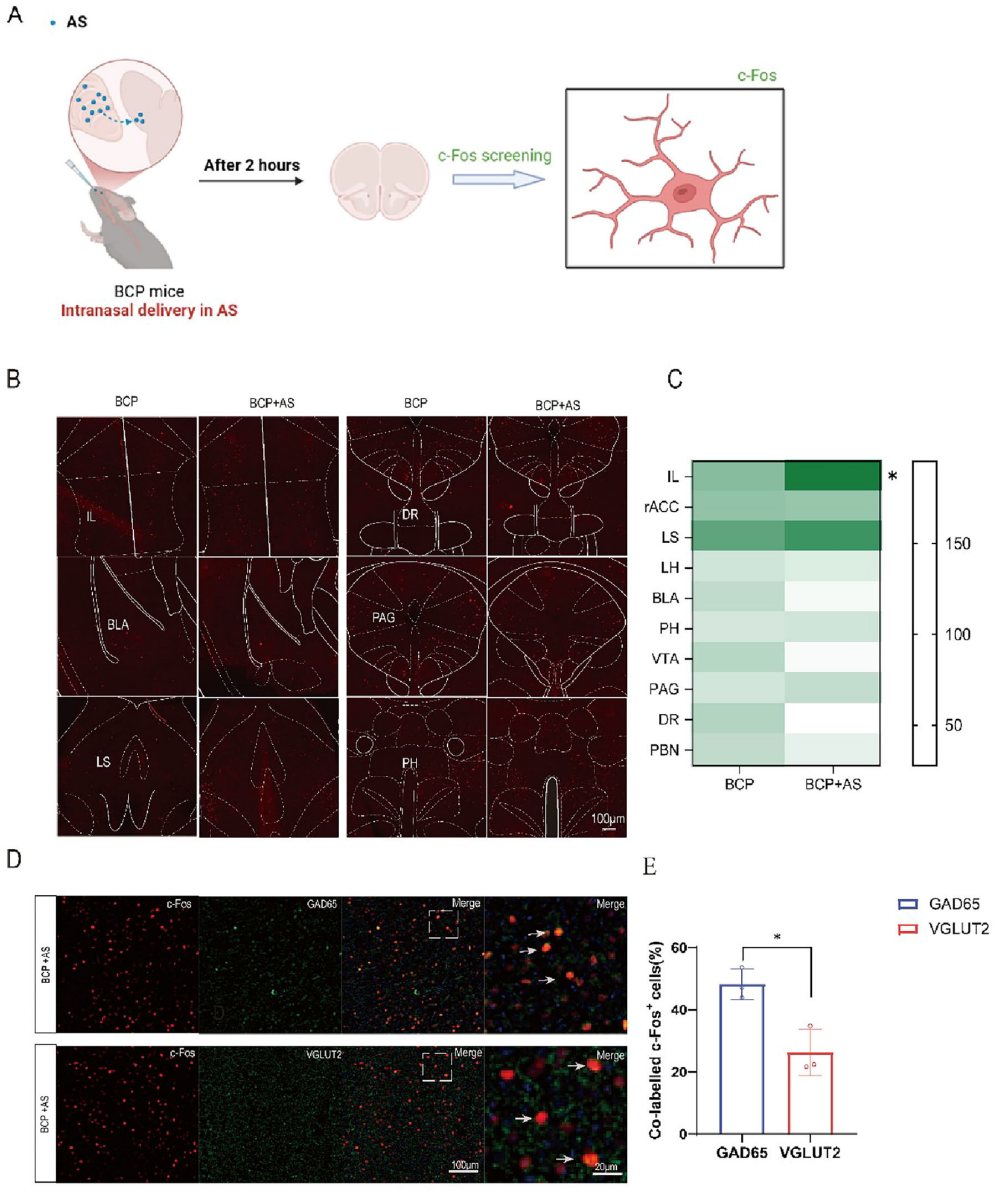
**A asticoside‐responsive antinociceptive ensemble in the BCP**. (A) The experimental diagram shows that in D21 mice with bone cancer pain, c‐Fos staining was detected 2 h after intranaside of asiaticoside. (B) Representative images of c‐Fos+ (red) cell immunofluorescence. Scale bar, 100 µm. (C) Quantification of c‐Fos positive neurons. Rostral anterior cingulate cortex (rACC); infralimbic cortex (IL); lateral septal (LS); lateral hypothalamic area (LH); basolateral amygdaloid (BLA); posterior hypothalamic area (PH); ventral tegmental area (VTA); periaqueductal gray (PAG); dorsal raphe nucleus (DR), parabrachial nucleus (PBN), (IL, *p < 0.05, BCP vs. BCP+AS group). (D) Immunofluorescence of GAD65 and VGLUT2 in the IL region. (E) Quantification of Co‐labelled c‐Fos‐positive neurons. Error bars are represented as mean ± SEM. *n* = 3 per group.

The expression of the activity‐induced immediate‐early gene, c‐Fos, serves as an indicator of neuronal activation. In this study, to pinpoint the specific brain region involved in the impaired cancer pain induced by asiaticoside, we comprised two groups: one treated with vehicle control and the other with asiaticoside prior to treatment (Figure 3A). Whole‐brain comparative analysis revealed alterations in neuronal activation across multiple brain regions, including the IL, VTA, DR, and BLA (Figure 3B,C). Notably, quantitative analysis of c‐Fos‐positive neurons demonstrated that the IL exhibited a more pronounced activation response with statistically significant differences following asiaticoside administration (Figure 3C). Furthermore, immunohistochemical analysis revealed that, in the IL region, the majority of activated neurons were GABAergic. (Figure 3D,E).

### 3.4 GABAergic Not Glutamatergic May Participate in Alleviating Cancer‐Induced Bone Pain

**FIGURE 4:**
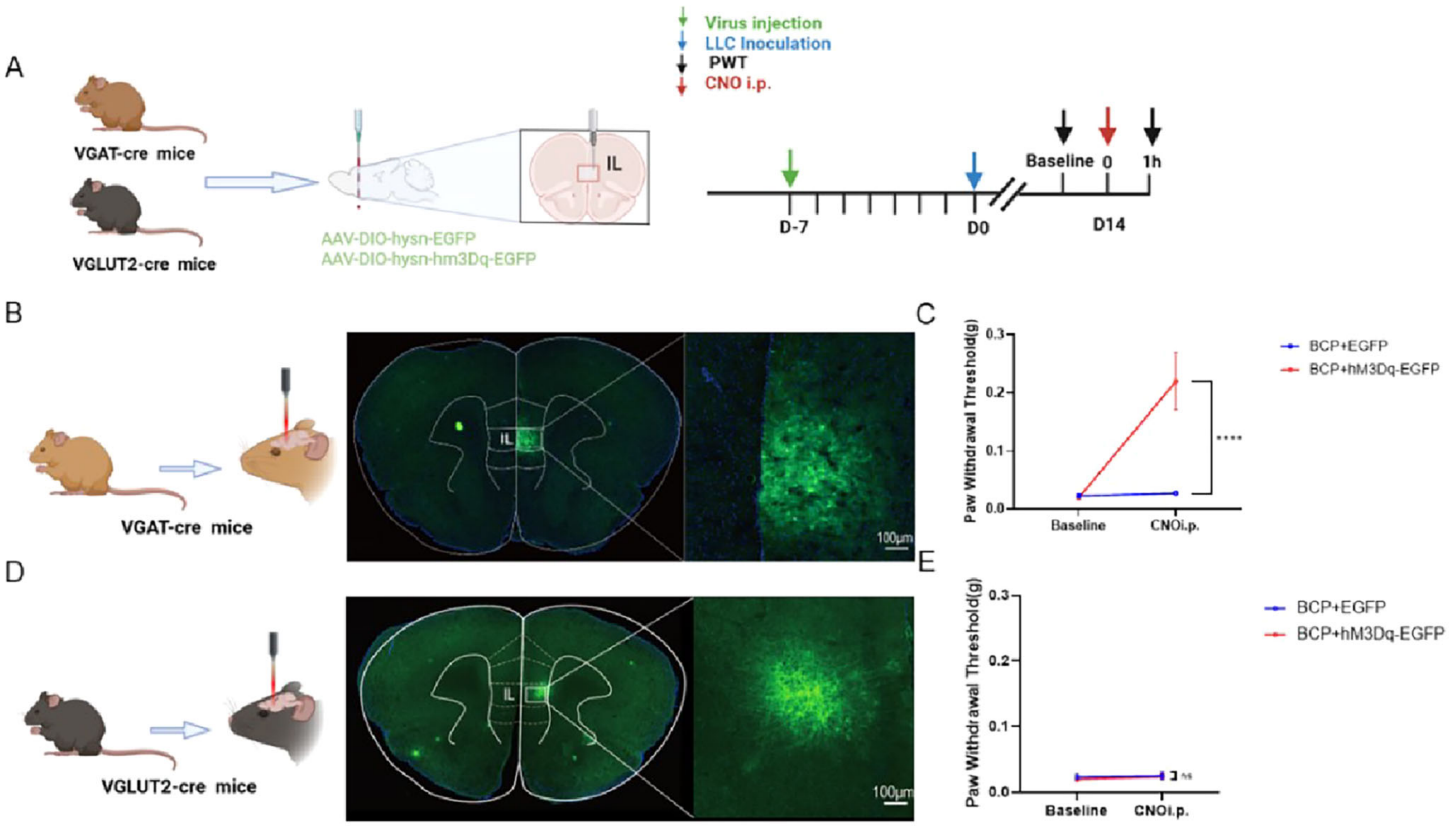
**GABAergic, but not Glutamatergic, neurons may participate in alleviating cancer‐induce bone pain**. (A) Experimental design. AAV2/9‐DIO‐hysn‐hM3Dq‐EGFP was injected in the IL region of VGAT‐cre mice and VGLUT2‐cre mice. Seven days after the AAV injection, tumor inoculation was established, followed by CNO administration on Day 14 after the tumor inoculation. (B) Representative image of EGFP expression in IL region of VGAT‐cre mice. Scale bar, 100 µm, (C) Paw withdrawal thresholds after the CNO injection of VGAT‐cre mice. (D) Representative image of EGFP expression in IL region of VGLUT2‐cre mice. Scale bar, 100 µm. (E) Paw withdrawal thresholds after the CNO injection of VGLUT2‐cre mice. ****p < 0.0001 BCP+EGFP versus BCP+hM3Dq+EGFP group, Error bars are represented as mean ± SEM. *n* = 5 per group.

To better validate whether GABAergic or glutamatergic neurons exert an analgesic effect, we injected Cre‐dependent viral constructs (AAV2/9‐hsyn‐DIO‐hM3Dq‐EGFP) or a control construct (AAV2/9‐hsyn‐DIO‐EGFP) into the IL of VGAT‐cre or VGLUT2‐cre mice (Figure 4A). Rapid and selective activation of GABAergic or glutamatergic neurons in the IL was induced by an intraperitoneal injection of CNO (1 mg/kg). We examined PWT before and 1 h after the CNO injection. The intraperitoneal injection of CNO into VGAT: hM3Dq mice activated GABAergic neurons in the IL region and increased PWT (Figure 4B,C). In contrast, activation of glutamatergic neurons in the IL region by injection of CNO into VGLUT2:hM3Dq mice did not produce an analgesic effect (Figure 4D,E). These results suggested that GABAergic, rather than Glutamatergic, neurons may participate in alleviating CIBP.

### 3.5 An Asiaticoside‐Responsive Antinociceptive Ensemble May Target the GABAergic Neuron in the IL Region

**FIGURE 5:**
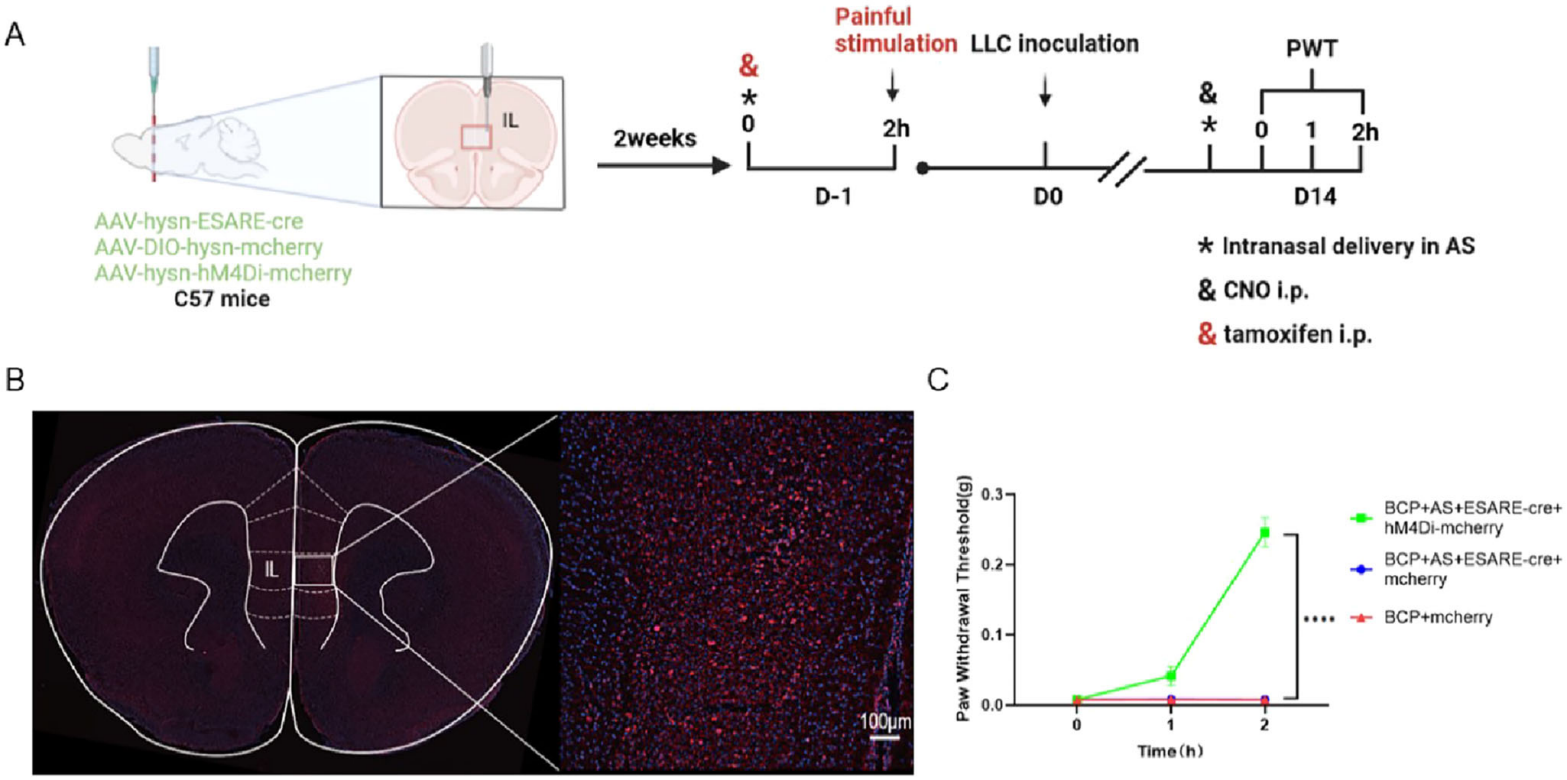
**A asiaticoside‐responsive antinociceptive ensemble may target the GABAergic neuron in the IL** (A) Experimental design. AAV2/9‐hysn‐ESARE‐cre and AAV2/9‐hysn‐DIO‐hM4Di‐mCherry was injected in the IL region of C57 mice. After 14 days after the AAV injection, asiaticoside and tamoxifen was administrated, PWT was established after 2 h to label AS activated neurons related to pain. And then tumor was injected into the femur, 14 days later, CNO was used to activate the hM4Di. (B) Schematics of AAV injections. Representative image of mCherry expression in IL region. Scale bar, 100 µm, (C) Paw withdrawal thresholds after the CNO injection. ****p < 0.0001 versus BCP+mCherry, Error bars are represented as mean ± SEM. *n* = 8 per group.

Lastly, to figure out the analgesic effect of asiaticoside is targeting the IL region, we injected enhanced Synaptic Activity Response Element (E‐SARE)—cre and AAV‐DIO‐hM4Di into IL aimed to label AS‐activated neurons related to pain and specifically carry out chemogenetic inhibition on these neurons (Figure 5A). First, we injected tamoxifen and stimulated mice with pain to pinpoint the specific cell populations responsible for related pain by asiaticoside. After establishing a mouse BCP model, we injected CNO to inhibit the analgesic effect of asiaticoside on neurons, which can significantly reverse the therapeutic effect of asiaticoside in BCP (Figure 5B,C). As such, we speculated that the asiaticoside‐responsive antinociceptive ensemble may target the GABAergic neuron in the IL region.

## 4 Discussion

This study demonstrates asiaticoside's antinociceptive efficacy in BCP through IL GABAergic modulation. c‐Fos staining revealed preferential IL activation following asiaticoside treatment. Activation of GABAergic neurons, rather than glutamatergic neurons, in the IL region, may alleviate CIBP. Meanwhile, inhibition of the asiaticoside‐activated neurons related to pain in the IL can reverse the pain relief, which indicates that the asiaticoside administered had an antinociceptive effect on BCP, which may target the Infralimbic GABAergic. Overall, this study provides strong evidence that asiaticoside can exert a protective effect on alleviating BCP and can provide a reference and basis for the treatment of clinical BCP.

Most recently, Asiaticoside has been shown to have anti‐inflammatory effects (He et al. 2024) in various neurodegenerative diseases, such as Parkinson's disease (He et al. 2024) and vascular dementia (Guo et al. 2021). Notably, Asiaticoside has been evidenced to alleviate pain symptoms after spinal cord injury through its microglia antioxidant and anti‐inflammatory effects (Ma et al. 2019). Consistent with prior findings, our study demonstrated that intragastric administration of high‐dose asiaticoside (60 and 120 mg/kg) significantly alleviates mechanical allodynia in the BCP model (Figure S1).

Based on the pharmacokinetic profile of asiaticoside in this study, which reaches its peak effect within 2 h, it exhibits similarities to currently available fast‐acting analgesic drugs, such as opioids (Plante and VanItallie 2010). These rapid‐onset analgesics typically exert their effects by directly modulating neuronal activity rather than neuroglial cells. To explore this possibility further, we used c‐Fos, an activity‐induced immediate‐early gene widely recognized as a marker of neuronal activation. By performing c‐Fos staining across the entire brain, we aimed to identify neurons activated by asiaticoside that are potentially involved in pain modulation. Compared to BCP mice, asiaticoside administration resulted in a significant increase in c‐Fos expression specifically within the IL region, which indicated that asiaticoside may activate the neuron in the IL region to exert an analgesic effect on BCP.

Several studies have explored the connection between IL activation and chronic pain. For instance, Yi et al. found that dysfunction of the IL acts as a central driver of spontaneous pain persistence in chronic inflammatory pain models (Ma et al. 2024b, Ma et al. 2019). IN addition, activation of afferent pathways from the IL to the PrL was shown to alleviate thermal hyperalgesia and mechanical allodynia in chronic inflammatory pain (Ma et al. 2024a). Our research in a BCP model also suggests that the analgesic effects of AS may be related to its activation of IL. More importantly, Li and colleagues demonstrated that optogenetic activation of IL neurons reduced c‐Fos expression in the CeLC and alleviated both mechanical and thermal pain in the opioid‐induced hyperalgesia (OIH) model. Opioid analgesics, commonly used for BCP patients, can lead to OIH (Cui et al. 2024). Given that AS alleviates pain by activating the IL brain region, it is possible that AS could not only be used to relieve BCP but also to improve the quality of life for BCP patients suffering from OIH.

The IL is primarily composed of glutamatergic neurons and GABAergic neurons. To further pinpoint the specific cell populations responsible for the analgesic effect of asiaticoside, we used immunohistochemistry to reveal the cell types of the activated neuron. The results showed that the percentage of GABAergic neurons among the c‐Fos‐positive cells was higher than that of glutamatergic neurons. To further confirm that asiaticoside alleviates BCP through the activation of GABAergic neurons, we employed chemogenetic methods to selectively activate or inhibit glutamatergic and GABAergic neurons in the IL region. The results from the chemogenetic experiments demonstrated that the activation of GABAergic neurons by asiaticoside effectively alleviates pain.

Altogether, all of these indicated the protective effect of asiaticoside on BCP, while further tests are necessary to provide a more comprehensive picture of the wide application of asiaticoside in clinically managing BCP. This study also has limitations. The neural circuits underlying IL modulation of pain may involve brain areas such as the PrL, amygdala (Likhtik et al. 2008), and the ventrolateral periaqueductal gray (Huang et al. 2019). While our study has identified IL GABAergic neurons as critical mediators of AS‐induced analgesia, their downstream neural circuits in BCP remain to be fully elucidated. Using anterograde tracing with AAV2/1‐Vgat‐cre injected into the IL, we identified prominent GABAergic projections to the lateral septum (LS), lateral hypothalamus (LH), and dorsal raphe (DR) brain regions previously implicated in pain modulation (Figure S2). These anatomical findings suggest that IL GABAergic neurons may exert their analgesic effects through these downstream targets. However, functional validation will be required to establish the causal contribution of each of these pathways to AS‐mediated analgesia. This aspect was not explored in the current study and requires further investigation. In addition, the exact mechanism by which asiaticoside affects GABAergic neurons in the IL needs to be further explored.

## Author Contributions

**Xin Li**: writing–original draft, data curation, writing–review and editing. **Jiayu Tong**: data curation, writing–review and editing. **Xinru Yuan**: resources. **Xiaoxuan Zhang**: data curation. **Haonan Yu**: data curation. **Chunlei Xing**: resources. **Jingxiang Wu**: funding acquisition. **Yuwei Qiu**: writing–review and editing. **Xingji You**: writing–original draft, writing–review and editing.

## Ethics Statement

The study protocol was approved by the Animal Care and Use Committee of Shanghai Chest Hospital, Shanghai Jiao Tong University [permission no. KS (Y)20186]

## Consent

The authors have nothing to report.

## Conflicts of Interest

The authors declare no conflicts of interest.

### Peer Review

The peer review history for this article is available at https://publons.com/publon/10.1002/brb3.70555

## Supporting information



Supplement Figure 1. Asiaticoside alleviated the cancer induced bone pain. *p < 0.01, **p<0.001 *vs*. BCP group, ^#^p < 0.01, ^##^p<0.001, *vs*. BCP+AS (60 mg/kg) group. Error bars are represented as mean ± SEM. n = 5 per group.
Supplement Figure 2. (A) The polysynaptic herpes simplex virus (HSV‐tdtomato) was injected into the IL region. Representative images showing the HSV‐tdtomato labeling in the infralimbic cortex (IL), Lateral septal nucleus (LS), piriform cortex (Pir), lateral hypothalamic area (LH), posterior hypothalamic area (PH). (B) The AAV2/1‐Vgat‐cre‐EGFP was injected into the IL Region. Representative images showing the GABAergic neurons of IL project to multiple brain regions, such as LS, LH, dorsal raphe nucleus (DR).

## Data Availability

The authors have nothing to report.
