# Supplementary material for: Infralimbic GABAergic May be the Target of Asiaticoside on Alleviating Bone Cancer Pain
Source: Brain Behav. 2025 May 19;15(5):e70555. doi: 10.1002/brb3.70555 (PMC12086296; doi:10.1002/brb3.70555)
Supplement: Supplementary file 1 — Supplement Figure 1. Asiaticoside alleviated the cancer induced bone pain. *p < 0.01, **p<0.001 vs. BCP group, #p < 0.01, ##p<0.001, vs. BCP+AS (60 mg/kg) group. Error bars are represented as mean ± SEM. n = 5 per group. Supplement Figure 2. (A) The polysynaptic herpes simplex virus (HSV‐tdtomato) was injected into the IL region. Representative images showing the HSV‐tdtomato labeling in the infralimbic cortex (IL), Lateral septal nucleus (LS), piriform cortex (Pir), lateral hypothalamic area (LH), posterior hypothalamic area (PH). (B) The AAV2/1‐Vgat‐cre‐EGFP was injected into the IL Region. Representative images showing the GABAergic neurons of IL project to multiple brain regions, such as LS, LH, dorsal raphe nucleus (DR). [file BRB3-15-e70555-s001.docx]

**Supplemental figure**

**
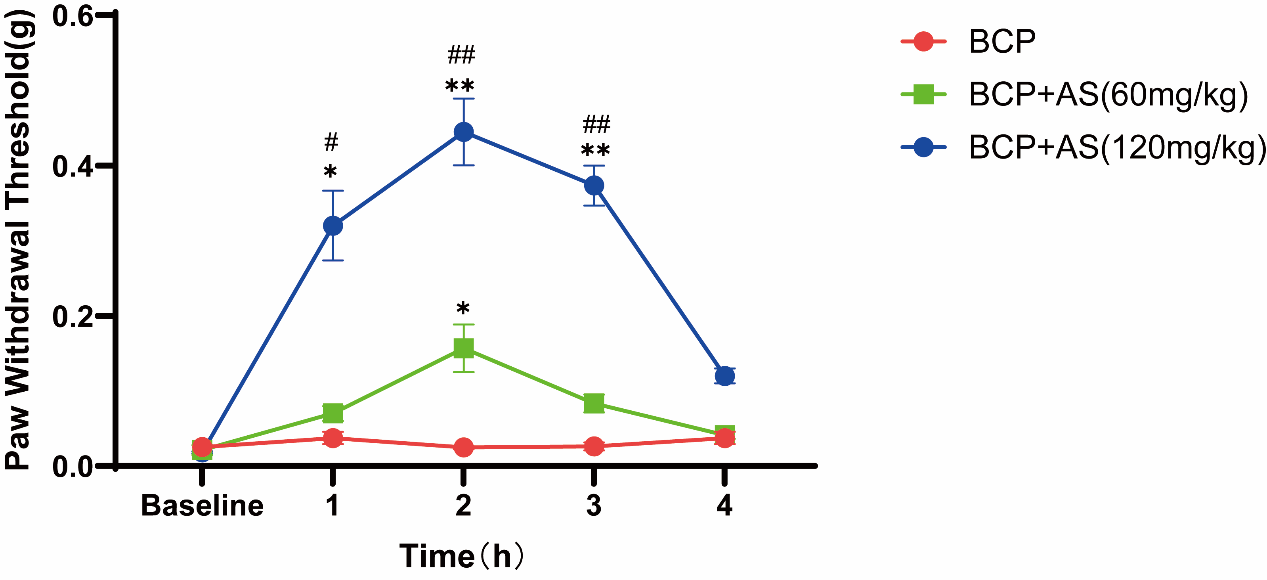
**

Supplement Figure 1. Asiaticoside alleviated the cancer induced bone pain. *p < 0.01, **p<0.001 *vs*. BCP group, ^#^p < 0.01, ^##^p<0.001, *vs*. BCP+AS (60mg/kg) group. Error bars are represented as mean ± SEM. n=5 per group.


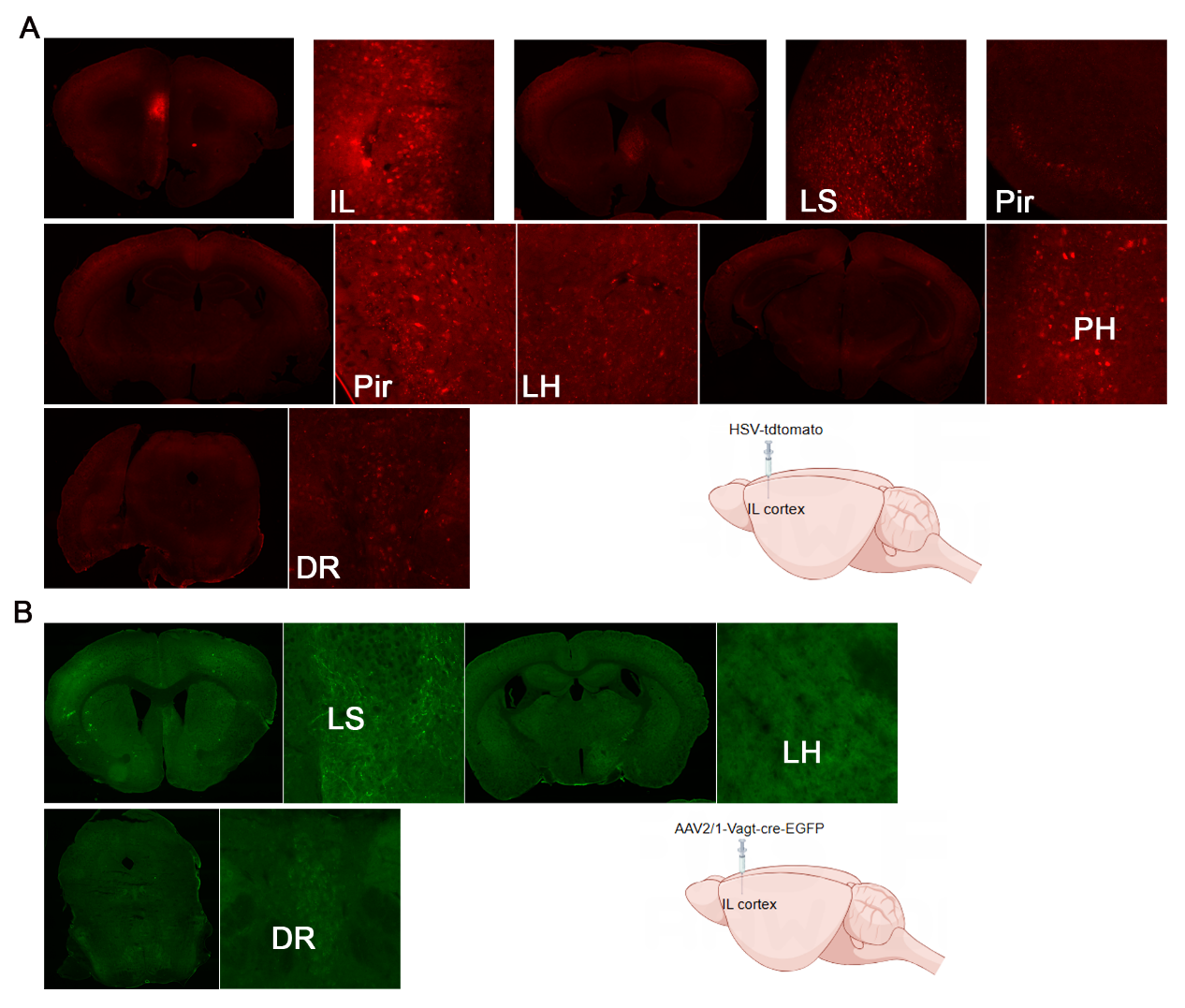


Supplement Figure 2. (A) The polysynaptic herpes simplex virus (HSV-tdtomato) was injected into the IL region. Representative images showing the HSV-tdtomato labeling in the infralimbic cortex (IL), Lateral septal nucleus (LS), piriform cortex (Pir), lateral hypothalamic area (LH), posterior hypothalamic area (PH). (B) The AAV2/1-Vgat-cre-EGFP was injected into the IL Region. Representative images showing the GABAergic neurons of IL project to multiple brain regions, such as LS, LH, dorsal raphe nucleus (DR).
